# A potent truncated form of human soluble CR1 is protective in a mouse model of renal ischemia–reperfusion injury

**DOI:** 10.1038/s41598-021-01423-y

**Published:** 2021-11-08

**Authors:** Anjan K. Bongoni, Ingela B. Vikstrom, Jennifer L. McRae, Evelyn J. Salvaris, Nella Fisicaro, Martin J. Pearse, Sandra Wymann, Tony Rowe, Adriana Baz Morelli, Matthew P. Hardy, Peter J. Cowan

**Affiliations:** 1 Immunology Research Centre, St. Vincent’s Hospital, Melbourne, PO Box 2900, Fitzroy, VIC 3065 Australia; 2 CSL Limited, Melbourne, VIC 3052 Australia; 3 CSL Behring AG, 3014 Bern, Switzerland; 4 Department of Medicine, University of Melbourne, Melbourne, VIC 3052 Australia

**Keywords:** Immunology, Physiology, Diseases, Nephrology, Pathogenesis

## Abstract

The complement system is a potent mediator of ischemia–reperfusion injury (IRI), which detrimentally affects the function and survival of transplanted kidneys. Human complement receptor 1 (HuCR1) is an integral membrane protein that inhibits complement activation by blocking the convertases that activate C3 and C5. We have previously reported that CSL040, a truncated form of recombinant soluble HuCR1 (sHuCR1), has enhanced complement inhibitory activity and improved pharmacokinetic properties compared to the parent molecule. Here, we compared the capacity of CSL040 and full-length sHuCR1 to suppress complement-mediated organ damage in a mouse model of warm renal IRI. Mice were treated with two doses of CSL040 or sHuCR1, given 1 h prior to 22 min unilateral renal ischemia and again 3 h later. 24 h after reperfusion, mice treated with CSL040 were protected against warm renal IRI in a dose-dependent manner, with the highest dose of 60 mg/kg significantly reducing renal dysfunction, tubular injury, complement activation, endothelial damage, and leukocyte infiltration. In contrast, treatment with sHuCR1 at a molar equivalent dose to 60 mg/kg CSL040 did not confer significant protection. Our results identify CSL040 as a promising therapeutic candidate to attenuate renal IRI and demonstrate its superior efficacy over full-length sHuCR1 in vivo.

## Introduction

Ischemia reperfusion (IR) injury (IRI) occurs in a variety of medical settings including hypotension, myocardial infarction, sepsis, and stroke^1–4^. In kidney transplantation, some degree of IRI, the most important cause of delayed graft function (DGF), is unavoidable and depending on severity it can impact negatively on both short- and long-term graft survival^5^. Renal IRI induces innate and adaptive immune responses and causes complement activation, cell apoptosis, platelet aggregation, microvascular dysfunction, and cellular tissue infiltration, which lead to acute kidney injury (AKI)^6^. The pathophysiological mechanisms that contribute to IRI are complex and incompletely understood. No specific clinical therapy to mitigate IRI is available to date, although the US Food and Drug Administration (FDA) recently granted orphan drug designation to two drugs (dimethyltryptamine and QPI-1002) for the prevention of DGF in kidney transplantation.

The activation of the complement system plays a critical role in the pathogenesis of renal IRI^7–10^. Complement has three distinct initiating pathways (classical, lectin, alternative), which converge to activate a common terminal pathway that results in the release of the anaphylatoxins C3a and C5a and the formation of the cytolytic C5b-9 membrane attack complex (MAC). These biologically active complement products enhance chemotaxis, promote phagocytosis, increase cell activation and lysis, and induce tissue damage^11^. Complement activation on intact self-cells is tightly regulated by several circulating and membrane-bound complement regulatory proteins. The latter include complement receptor type 1 (CR1, CD35), decay-accelerating factor (DAF, CD55) and membrane cofactor protein (MCP, CD46)^12^. Clinical and experimental studies have shown that loss of complement regulators and increased local production of complement contribute to unregulated complement activation during renal IRI^13,14^. In clinical renal transplant biopsies, rejection was strongly correlated with extensive peritubular deposition of the complement split products C4d and C3d^15^. In mice, a deficiency in complement components (C3, C5, C6, or Factor B) or C3a and/or C5a receptors protected against renal IRI^13,16,17^, whereas mice deficient in complement regulators were more susceptible^18,19^. Therefore, complement activation at multiple stages contributes to renal IRI. However, despite several attempts in recent years^9,20–22^, the effective clinical therapy of renal IRI by attenuating complement activation has not been reported.

Human CR1 (HuCR1), also known as the C3b/C4b receptor, is a multi-functional polymorphic glycoprotein expressed on the surface of erythrocytes, monocytes, neutrophils, B cells, some T cells, follicular dendritic cells, and glomerular podocytes^23^. The complement regulatory activity of HuCR1 is contained within its 1971-amino acid extracellular domain, which is composed of four long homologous repeats (LHR-A through -D) with a total of 30 short consensus repeats (SCRs)^24^. A soluble form of HuCR1 containing the extracellular domain but lacking the transmembrane and cytoplasmic domains is present in the circulation at extremely low concentrations, most likely as a by-product of proteolytic shedding^25^. Both membrane-bound and soluble CR1 regulate complement activity by accelerating the decay of the C3 and C5 convertases and acting as a cofactor for the serine protease factor I, which degrades C3b and C4b^26^. A recombinant form of soluble HuCR1 (sHuCR1) known as TP10 has previously been tested for its efficacy against complement-mediated pathologies. Studies using animal models demonstrated that treatment with TP10 had beneficial effects in arthritis^27^, hyperacute rejection^28^, IRI of small intestine^29^ and skeletal muscle^30^, tissue injury and burns^31^. However, TP10 has a relatively short half-life^32,33^ and failed to meet primary end points in clinical trials^33,34^. Recently, we have developed CSL040, a sialylated truncated variant of sHuCR1 lacking the LHR-D domain. CSL040 exhibits higher complement regulatory activity and an extended half-life compared with sHuCR1^35^, and has shown benefit in a model of antibody-mediated glomerulonephritis in mice^35^. These data led us to further investigate the efficacy of CSL040 in a mouse model of warm renal IRI.

## Results

### Complement activation in renal IRI

**Figure 1:**
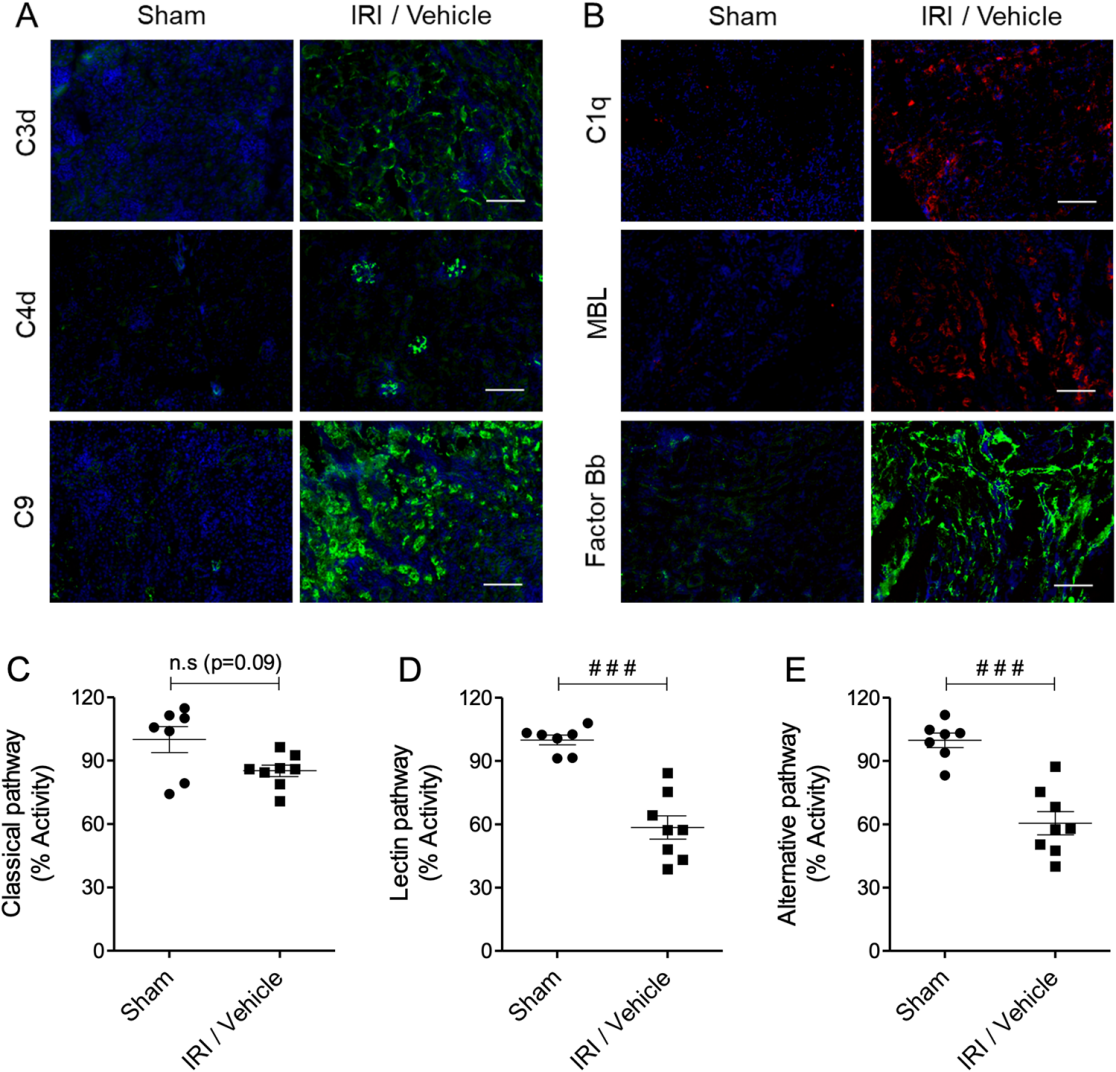
Analysis of complement deposition in renal IRI. Mice were subjected to either right nephrectomy only (sham) or right nephrectomy, 22 min left kidney ischemia and 24 h reperfusion (IRI/vehicle). (**A**,**B**) Representative images of kidney deposition of (**A**) complement activation markers C3d, C4d and C9, and (**B**) pathway-specific components C1q (classical), MBL (lectin) and factor Bb (alternative) assessed by immunofluorescence staining/confocal microscopy. Scale bar: 130 μm. (**C**–**E**) Twenty-four hours after reperfusion, serum samples were collected and analysed for complement consumption by measuring (**C**) classical, (**D**) lectin, and (**E**) alternative pathway activities using pathway-specific ELISA kits. Statistical analysis was performed with Mann–Whitney U test ^(###^p < 0.001). The data shown are mean ± SEM, n = 7–8.

In our mouse model of unilateral warm renal IRI, the right kidney is removed, the left renal pedicle is clamped for 22 min, and kidney injury and function are assessed 24 h post-reperfusion. The first set of experiments focused on validation of the complement pathway as a target in this model. For this purpose, kidneys were examined for the deposition of complement activation markers by immunofluorescence staining/confocal microscopy, and serum was collected to measure consumption of complement components. Compared to sham controls, mice undergoing kidney IRI exhibited significant deposition of C3d, C4d and C9 on the basement membrane of tubular epithelium and on vascular and glomerular endothelium (Fig. 1A). C3d is a marker for complement activation by any of the three pathways, while C4d indicates engagement of the classical and/or lectin pathways, and C9 indicates formation of the MAC, the final step of complement activation. To further investigate which complement pathways were activated, kidneys were stained for the pathway-specific components C1q (classical), MBL (lectin) and factor Bb (alternative). Deposition of all 3 markers was evident in IRI kidneys (Fig. 1B). Serum collected from the mice at 24 h was analysed for the activity of each pathway. Lectin and alternative pathway activities were significantly reduced in IRI versus sham mice (Fig. 1D–E; both p < 0.001), indicating consumption of components in those pathways. Classical pathway activity was also reduced in IRI mice, although this did not reach statistical significance (Fig. 1C; p = 0.09). Together these data suggest activation of the lectin and alternative complement pathways, and to a lesser extent of the classical complement pathway, in the warm renal IRI model.

### Treatment with CSL040 reduced IR-induced renal injury

**Figure 2:**
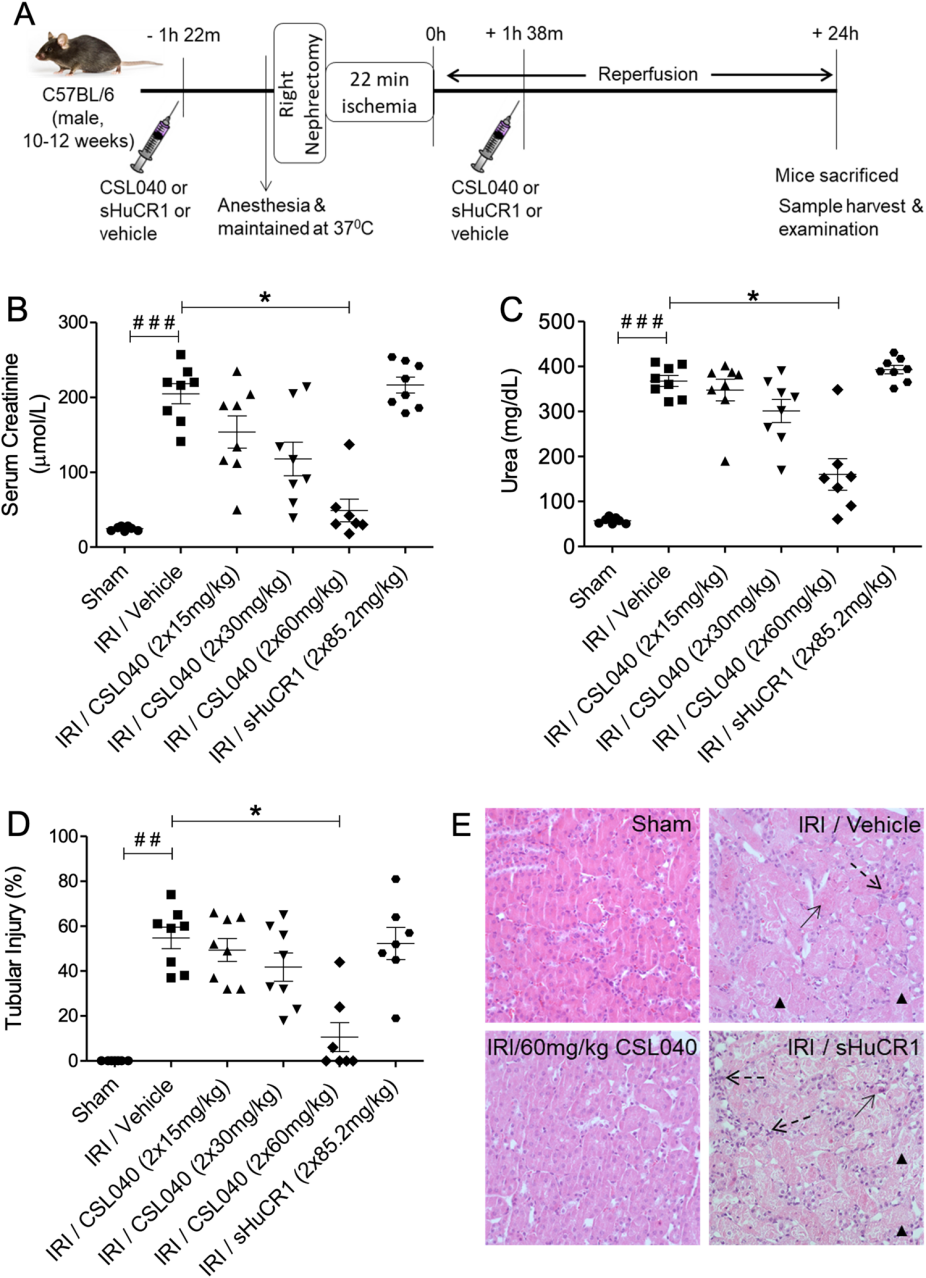
Protection against IR-induced renal dysfunction by CSL040 treatment. (**A**) A schematic illustration of the experimental setup and time course of the mouse model. Mice were treated with two doses of vehicle control, 15–60 mg/kg CSL040, or 85.2 mg/kg sHuCR1 as indicated 1 h prior to ischemia and 3 h after the first injection. Twenty-four hours after reperfusion, renal function was assessed by measuring (**B**) serum creatinine and (**C**) urea, and (**D**) tubular injury in kidney sections. (**E**) Representative images of hematoxylin–eosin (H&E) stained kidney sections used to score renal tubular injury (×400 magnification). Arrowheads indicate tubular injury, necrosis, swell and dilation; arrows indicate cast formation; dashed arrows indicate inflammatory cell infiltration. Significance was tested using Mann–Whitney U test (^##^p < 0.01, ^###^p < 0.001 for sham vs. IRI/vehicle), and One-way ANOVA Kruskal–Wallis and Dunn multiple comparisons test (*p < 0.05 for IRI/vehicle vs. all treatments). The data shown are mean ± SEM (n = 7–8 per group).

IRI/vehicle-treated mice showed significantly elevated serum creatinine and urea (both p < 0.001 versus sham) at 24 h, indicating a significant loss of renal function (Fig. 2A–C). Treatment with two doses of 15, 30 or 60 mg/kg CSL040 protected against loss of renal function in a dose-dependent manner, reaching statistical significance with the highest dose (p < 0.05 versus IRI/vehicle) (Fig. 2A–C). Histological analysis of kidneys from the IRI/vehicle group revealed extensive tubular injury (p < 0.001 versus sham) with necrosis, cell swelling, dilatation, cast formation, and overall disruption of renal architecture. Consistent with the functional readouts, CSL040 treatment dose-dependently reduced tubular injury, with significance at the highest dose of 60 mg/kg (p < 0.05 versus IRI/vehicle) (Fig. 2D,E). Treatment with two doses of 85.2 mg/kg full-length sHuCR1 (an equimolar dose to 60 mg/kg CSL040) did not confer significant protection against IR-induced renal dysfunction or tubular injury (Fig. 2B–E). CSL040 at the highest dose significantly reduced the number of cleaved caspase-3 positive apoptotic cells in the kidney, whereas full-length sHuCR1 did not (Supplementary Fig. 1).

### Treatment with CSL040 reduced renal IR-induced complement activation

**Figure 3:**
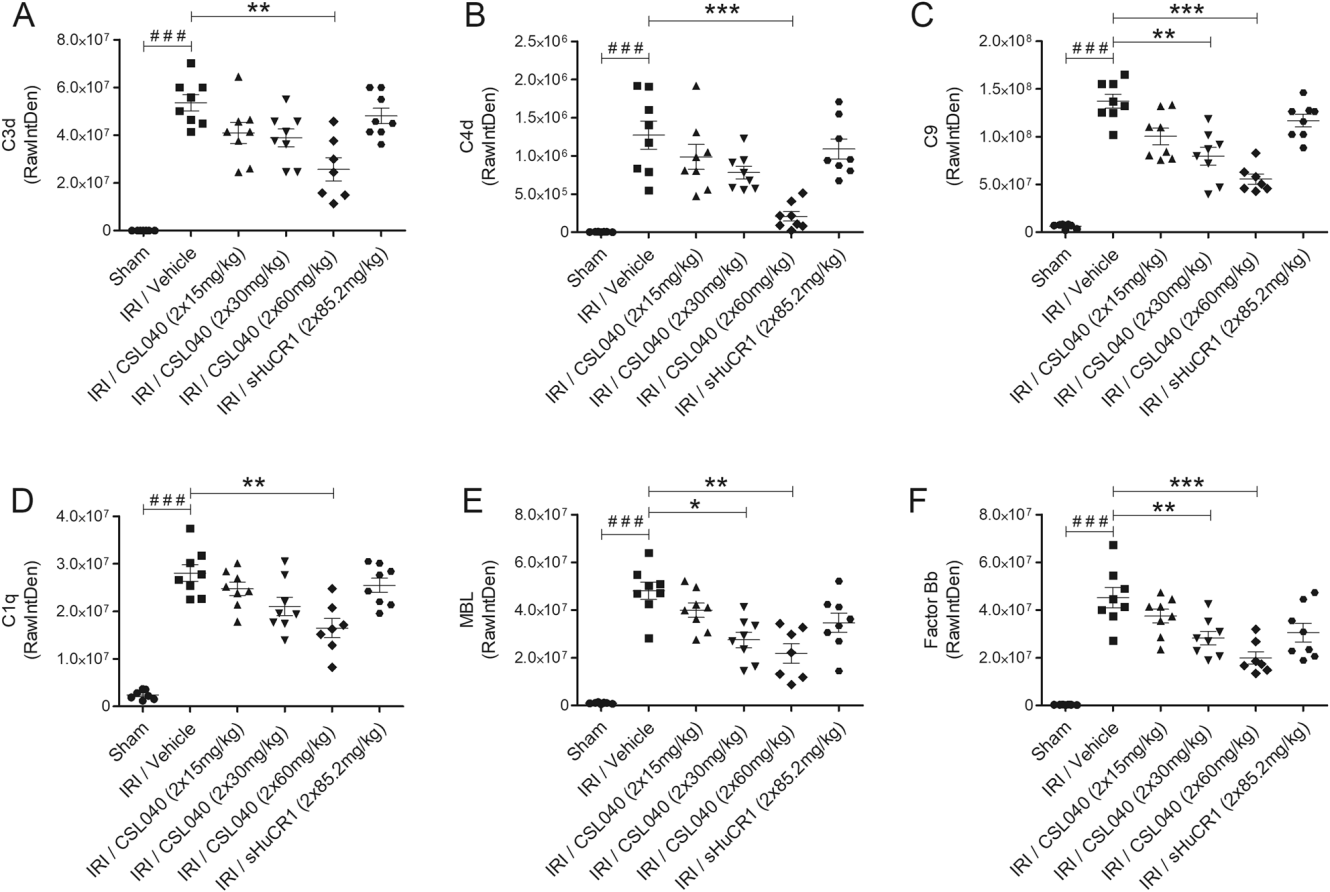
Reduction of IR-induced complement activation and tissue deposition by CSL040 treatment. Twenty-four hours after reperfusion, kidney sections were assessed for (**A**) C3d, (**B**) C4d, (**C**) C9, (**D**) C1q, (**E**) MBL, and (**F**) factor Bb deposition by immunofluorescence staining/confocal microscopy and image J analyses. Significance was tested using Mann–Whitney U test (^###^p < 0.001 for sham vs. IRI/vehicle), and One-way ANOVA Kruskal–Wallis and Dunn multiple comparisons test (*p < 0.05, **p < 0.01, ***p < 0.001). The data shown are mean ± SEM (n = 7–8 per group).

Quantitation of immunofluorescence staining of kidney sections demonstrated significantly higher intrarenal deposition of C3d, C4d, and C9 in IRI/vehicle-treated mice than in sham mice (Fig. 3A–C; all p < 0.001). CSL040 treatment dose-dependently reduced the levels of all 3 markers, reaching statistical significance at 60 mg/kg for C3d and C4d (Fig. 3A,B; C3d: p < 0.01, C4d: p < 0.001) and at 30 mg/kg for C9 (Fig. 3C; 30 mg/kg: p < 0.01, 60 mg/kg: p < 0.001). Treatment with full-length sHuCR1 did not significantly reduce deposition of C3d, C4d or C9 (Fig. 3A–C).

A similar pattern was observed for the pathway-specific markers C1q, MBL and factor Bb. Mice from the IRI/vehicle group showed significant intrarenal deposition of all 3 markers compared to sham mice (Fig. 3D–F; all p < 0.001), confirming the activation of the classical, lectin and alternative pathways in this model. CSL040 at 60 mg/kg significantly reduced C1q (p < 0.01), and at 30 mg/kg for MBL (30 mg/kg: p < 0.05, 60 mg/kg: p < 0.01) and factor Bb (30 mg/kg: p < 0.01, 60 mg/kg: p < 0.001); full-length sHuCR1 also caused a reduction, but this did not reach statistical significance for any of the markers (Fig. 3D–F).

**Figure 4:**
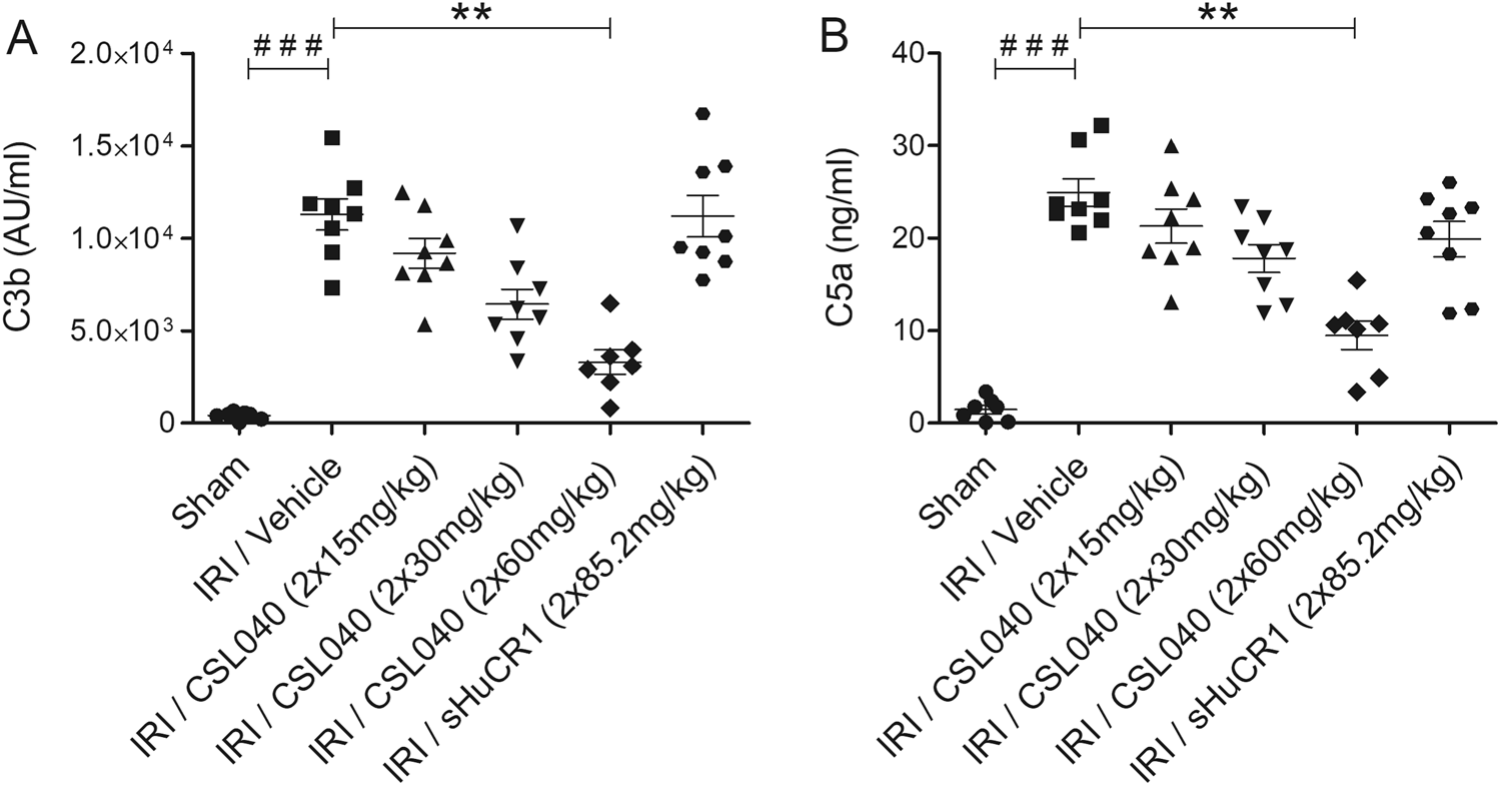
Attenuation of IR-induced systemic complement activation products C3b and C5a by CSL040 treatment. Plasma samples collected 24 h after reperfusion were analysed for (**A**) C3b, and (**B**) C5a by ELISA. Significance was tested using Mann–Whitney U test (^###^p < 0.001 for sham vs. IRI/vehicle), and One-way ANOVA Kruskal–Wallis and Dunn multiple comparisons test (**p < 0.01 for IRI/vehicle vs. all treatments). The data shown are mean ± SEM (n = 7–8 per group).

Complement activation was assessed at the systemic level by measuring circulating C3b and C5a. In line with the histological analyses, serum C3b and C5a were significantly increased in IRI/vehicle mice compared to sham mice (Fig. 4A,B; both p < 0.001), and significantly reduced by treatment with 60 mg/kg CSL040 (Fig. 4A,B; both p < 0.01 versus IRI/vehicle). Full-length sHuCR1 at an equimolar dose did not significantly reduce C3b or C5a in serum (Fig. 4A,B).

### Treatment with CSL040 attenuated renal IR-induced endothelial cell activation and glycocalyx shedding

**Figure 5:**
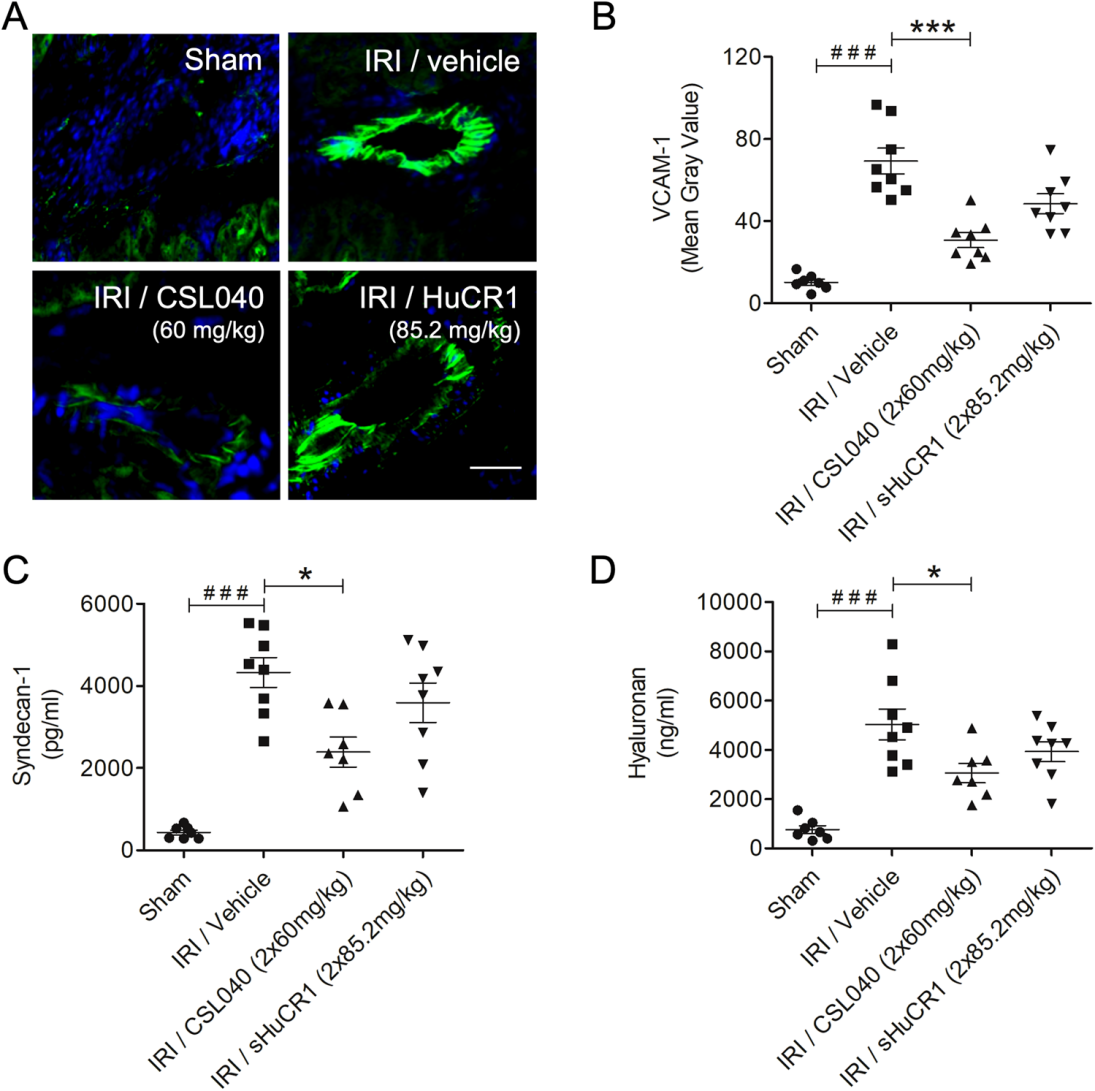
Inhibition of IR-induced endothelial activation and glycocalyx shedding by CSL040 treatment. (**A**) Representative immunofluorescence images of VCAM-1 expression on kidney Sects. 24 h after reperfusion. Scale bar: 30 μm. (**B**) Quantitative analysis of endothelial expression of VCAM-1 by image J analysis. (**C**,**D**) Plasma levels of syndecan-1 and hyaluronan measured by ELISA. Significance was tested using Mann–Whitney U test (^###^p < 0.001 for sham vs. IRI/vehicle), and One-way ANOVA Kruskal–Wallis test and Dunn multiple comparisons test (*p < 0.05, ***p < 0.001 for IRI/vehicle vs. all treatments). The data shown are mean ± SEM (n = 7–8 per group).

Renal endothelial cell activation and glycocalyx degradation as a consequence of kidney IRI contribute to inflammation and loss of renal architecture. Activation of the renal endothelium was assessed by measuring tissue VCAM-1 expression. Expression of VCAM-1 was significantly upregulated in IRI/vehicle mice compared to sham mice (Fig. 5A,B; p < 0.001). Treatment with 60 mg/kg CSL040 significantly reduced VCAM-1 expression (Fig. 5A,B; p < 0.001 versus IRI/vehicle). The equimolar dose of full-length sHuCR1 also reduced VCAM-1 expression, but this was not statistically significant (Fig. 5A,B).

Plasma syndecan-1 and hyaluronan levels were measured to assess shedding of the endothelial glycocalyx. Both markers were significantly increased in IRI/vehicle mice compared to sham mice (Fig. 5C,D; both p < 0.001). Treatment with 60 mg/kg CSL040 significantly reduced the levels of circulating syndecan-1 and hyaluronan compared to IRI/vehicle (Fig. 5C,D; both p < 0.05 versus IRI/vehicle). Full-length sHuCR1 treatment had no effect on the levels of plasma syndecan-1 and hyaluronan (Fig. 5C,D). Together, these data suggest that inhibition of complement by CSL040 reduced IR-induced endothelial activation and glycocalyx degradation, thereby preserving endothelial integrity.

### Treatment with CSL040 attenuated renal IR-induced innate immune cell infiltration

**Figure 6:**
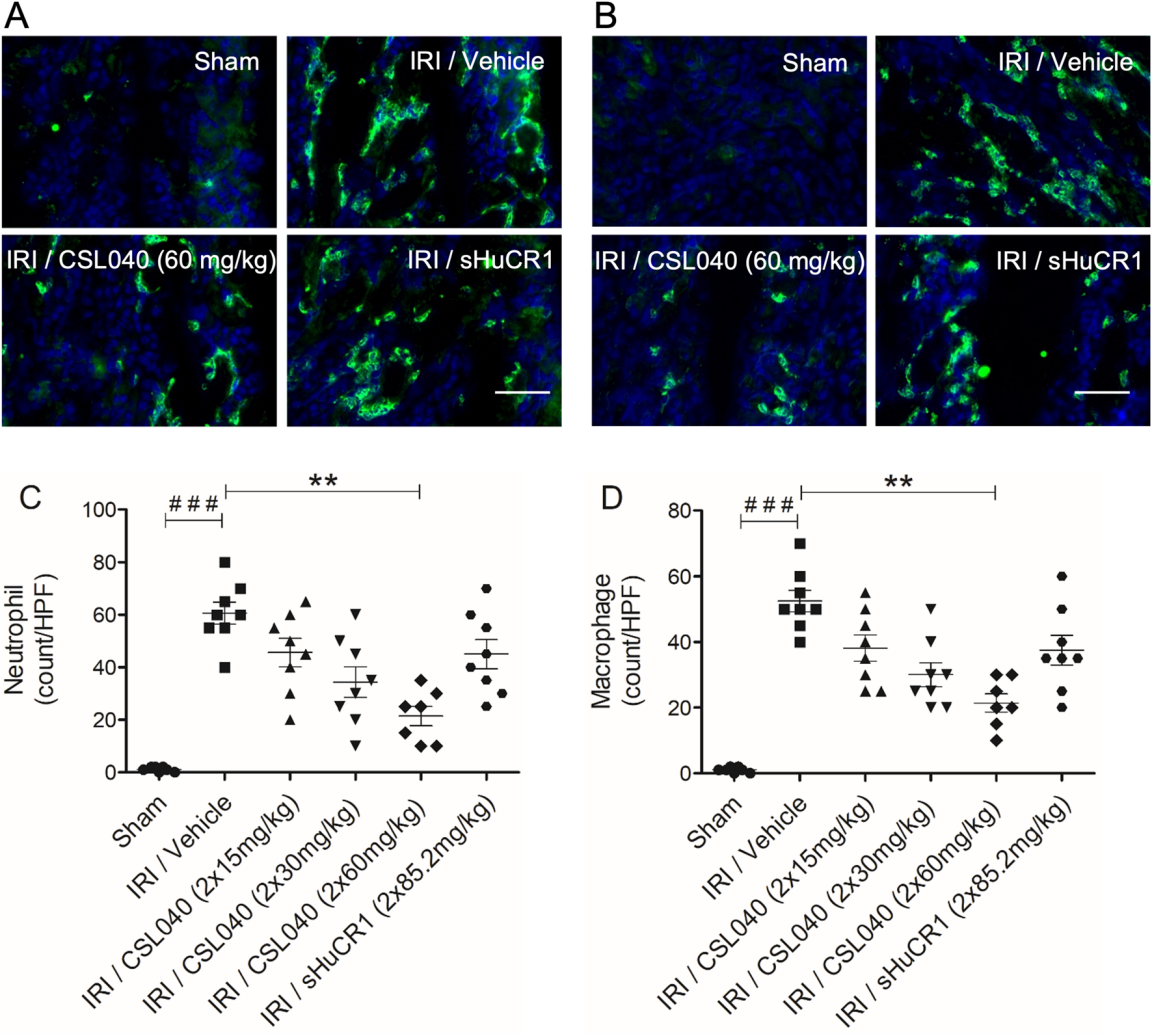
Reduction of IR-induced neutrophil and macrophage recruitment by CSL040 treatment. (**A**,**B**) Representative immunofluorescence images of neutrophil (Ly-6G positive) and macrophage (F4/80) kidney infiltration. Scale bar: 50 μm. (**C**,**D**) The numbers of neutrophil and macrophage infiltrate were expressed as count per high-power field (HPF), and quantitatively measured by image J analysis. Significance was tested using Mann–Whitney U test (^###^p < 0.001 for sham vs. IRI/vehicle), and One-way ANOVA Kruskal–Wallis and Dunn multiple comparisons test (**p < 0.01 for IRI/vehicle vs. all treatments). The data shown are mean ± SEM (n = 7–8 per group).

Infiltration by neutrophils and macrophages is a key feature of renal IRI-induced local inflammation^36^. Kidney sections were analysed for the presence of infiltrating neutrophils (Ly-6G+) and macrophages (F4/80+) by immunofluorescence staining/confocal microscopy. Renal IRI was associated with recruitment of both cell types to the tubular interstitium at the corticomedullary junction (Fig. 6A,B) which was significant for both populations compared to sham (Fig. 6C,D; both p < 0.001 IRI/vehicle versus sham). Treatment with CSL040 dose-dependently reduced the number of infiltrating neutrophils and macrophages following IR, reaching statistical significance with the highest dose of 60 mg/kg (Fig. 6C,D; both p < 0.01 versus IRI/vehicle). As observed for many of the other readouts, the equimolar dose of full-length sHuCR1 did not significantly affect macrophage or neutrophil recruitment/infiltration (Fig. 6C,D).

## Discussion

Transplant-related IRI is an unavoidable inflammatory response that happens in all transplanted organs during the process of organ retrieval and reperfusion in the recipient. In kidney transplantation, IRI is the predominant mechanism leading to organ dysfunction or DGF, which is associated with an increased risk of chronic rejection, and worse long-term graft function and survival^20,37,38^. To date, no approved treatments for IRI or DGF are available. There are abundant pre-clinical and clinical data demonstrating that unregulated complement activation contributes to the pathogenesis of a wide range of conditions including renal IRI and DGF^20,37–39^. It has been shown in animal models that deficiency in complement components or treatment with complement inhibitors resulted in significantly reduced renal IRI^7,9,16,17,40,41^. In this study, we identified CSL040, a truncated form of recombinant sHuCR1 with enhanced inhibitory activity and pharmacokinetic properties^35^, as a promising therapeutic candidate to attenuate complement activation and ameliorate renal IRI.

It is known that all complement activation pathways can be involved in the pathogenesis of renal IRI, DGF, and transplant rejection, but the precise mechanisms and the relative contribution of the individual pathways has been a subject of debate. Each pathway uses different triggers under various pathogenic conditions such as infection or ischemia. Determining which pathway(s) initiates the complement cascade in IRI may inform the development of more specific therapeutic strategies. Initial studies using C4- or factor B-deficient mice indicated a predominant role for the alternative pathway and lack of involvement of the classical and lectin pathways^42,43^. In contrast, the lectin pathway was implicated in a study using mice deficient in MBL-A and MBL-C^44^. There is also evidence to suggest that all 3 pathways are activated by renal IR in large animals including swine and humans^45,46^. To clarify which of the pathways was activated in our model, we stained for C1q, MBL, and factor Bb in injured kidneys. Deposition of all 3 markers was observed and was most pronounced for MBL and factor Bb. In line with this, analysis of complement functional activity in serum revealed that renal IRI induced higher consumption of the components of the lectin and alternative pathways than of the classical pathway. The injured kidneys also showed intense deposition of C3d, C4d, and C9, indicating full activation of complement.

In renal IRI, complement deposition occurs on the outer surface of the proximal tubules early after reperfusion^9,16^. Proximal tubular epithelial cells generate local C3 and express only low levels of complement regulatory proteins^13,47^, helping to explain the vulnerability of the kidney, particularly tubular cells, to complement attack following IRI. It has been suggested that intrarenal C3 synthesis may play a pathogenic role during kidney transplant rejection episodes^48^. A study of renal transplant patients showed a correlation between the level of C3 gene expression in donor kidneys at the time of implantation and the decline of graft function after transplantation^49^. C3 activation, the convergence point of all three complement pathways, is the central stage to generate nearly all complement effector products, including C3b. The formation of C3b is a prerequisite for the generation of C3 and C5 convertases and the formation of MAC, and its tissue deposition amplifies the complement-mediated disease pathophysiology^50^. Altogether, the published and present data suggest that multiple complement pathways are activated during reperfusion in renal IRI, and its severity depends critically on both early activation steps during reperfusion and on local C3 upregulation. Upstream complement inhibition at the level of C3/C3b or the C3 convertase has therefore been considered a promising approach. Currently no C3-targeted inhibitors are in clinical use for IRI and DGF, however, several promising candidates have emerged in recent years^9,51^. sHuCR1 has been investigated as a treatment for IRI of various organs including the kidney^31,52–54^, because it blunts the impact of C3 by accelerating the decay of the C3 and C5 convertases and promoting the factor I-mediated inactivation of C3b and C4b. TP10, a recombinant sHuCR1 produced in CHO cells, protected rat renal allografts from complement-mediated inflammatory injury^32^. TP10 has demonstrated modest efficacy in a clinical trial in which 59 patients undergoing lung transplantation received TP10 or placebo before reperfusion^34^. Although early postoperative deaths and the incidence of rejection episodes were not significantly different, the proportion of patients extubated at 24 h was significantly higher in the TP10 group^34^. This study confirmed the potential for sHuCR1 in the treatment of IRI. In the current study, we show that two doses of 60 mg/kg of CSL040, a sialylated and truncated form of sHuCR1 with enhanced complement inhibitory activity and an extended half-life^35^, effectively blocked all 3 pathways of complement activation and ameliorated renal IRI in mice. Treatment with an equimolar amount of full-length sHuCR1 with a similar level of sialylation to CSL040^35^ did not significantly reduce complement activation or renal dysfunction. It is important to note that the same sHuCR1 material was used in this and our earlier functional comparison with CSL040^35^, ruling out the possibility that the observed difference in efficacy was due to batch-to-batch variation in protein quality and activity. We conclude that CSL040 provides significant benefits over full-length sHuCR1 in protecting the kidney against ischemic injury.

Activation of complement can directly affect the function of the endothelium by inducing glycocalyx shedding, release of proinflammatory cytokines and chemokines, and upregulation of adhesion molecule expression. Endothelial dysfunction is concomitant with permanent loss of up to 50% of peritubular capillaries, interstitial fibrosis and tubular atrophy^55,56^. We have previously shown the rapid activation of the endothelium and degradation of the endothelial glycocalyx after renal IRI^10^. Disruption of the glycocalyx results in increased vascular permeability, interstitial edema, and loss of endothelial integrity and function^57^. In addition, breakdown components of the glycocalyx (syndecan-1, hyaluronan fragments, heparan sulfate, and biglycan) have been shown to activate innate and adaptive immunity^58^. In this study, we found that treatment with CSL040 attenuated endothelial activation and glycocalyx shedding. These results provide further evidence that complement activation is a critical effector mechanism of post-ischemic renal inflammation and injury. Treatment with full-length sHuCR1 showed no significant reduction in endothelial injury, consistent with its lower potency compared to CSL040. The activation of complement promotes tissue infiltration by leukocytes via chemokine expression and enhancement of adhesion molecule interactions and transmigration across the impaired vascular barrier^59^. The infiltrating leukocytes produce proinflammatory cytokines and oxygen radicals and release proteases and myeloperoxidase, aggravating injury^60^. As previously reported^9,10^, we found that IR induced infiltration of neutrophils and macrophages, particularly in the corticomedullary junction of the kidney. Treatment with CSL040 significantly inhibited their recruitment, confirming that complement activation is critical in this aspect of the injury process. In a separate study, we have demonstrated that interfering with neutrophil activation and homing, by antibody blockade of the G-CSF receptor (G-CSFR), is protective in our model of warm renal IRI^36^. Future studies will examine the possibility that CSL040 treatment and G-CSFR blockade, which target different mechanisms, may have complementary or synergistic protective effects.

In conclusion, we have demonstrated that CSL040 effectively protects against renal IRI, significantly reducing renal dysfunction, tubular injury, complement activation and deposition, endothelial activation and damage, and neutrophil and macrophage infiltration. This study identifies CSL040 as a potential therapeutic candidate to attenuate IRI and thereby improve graft function and survival in kidney and/or other solid organ transplants.

## Materials and methods

### Animals

Male C57BL/6 mice were purchased from the Animal Resources Centre (Canning Vale, Western Australia), and housed in an approved animal facility (Bioresources Centre, St. Vincent's Hospital Melbourne). All experiments were carried out with the approval of the Animal Ethics Committee (AEC Reference number: 019/17) of St. Vincent's Hospital Melbourne and in accordance with relevant guidelines and regulations. The animal studies are reported in compliance with the ARRIVE guidelines (Animal Research: Reporting in Vivo Experiments).

### Warm renal IRI

10–12-week-old male C57BL/6 mice were subjected to warm renal IRI as described previously^10^. Briefly, mice were anesthetized by intraperitoneal (i.p.) administration of ketamine (100 mg/kg) and xylazine (15 mg/kg) and core body temperature was kept at a constant 37 °C by placing a heat pad beneath the animal. Prior to surgery, 0.1 mg/kg of the analgesic buprenorphine was administered subcutaneously (s.c). Using midline abdominal incision, the kidneys were exposed and the renal pedicles were bluntly dissected. After right nephrectomy, the left renal pedicle was occluded with a microvascular clamp (Roboz, Rockville, MD) for 22 min at 37 °C in a temperature-controlled chamber. After clamp removal, the kidney was observed for the colour change indicative of even blood reflow. The surgical wound was then sutured, and mice received 8 ml/kg warm saline into the abdominal cavity prior to wound closure. Mice were recovered on a heat pad at 37 °C with atipamezole (0.25 mg/kg, s.c. injection) to hasten recovery from anaesthesia. 24 h after reperfusion, the mice were anesthetized and exsanguinated, and blood and kidney samples were obtained. Sham-operated mice had identical surgical procedures except that the left renal pedicle was not clamped.

A dose escalation study was performed using two doses of CLS040 in the range 15 to 60 mg/kg (in a volume of 200–220 μL), 85.2 mg/kg of sHuCR1 (molar equivalent dose to 60 mg/kg CSL040) or Phosphate Buffered Saline (PBS, vehicle control) were administered via i.p. injection 1 h prior to ischemia and 3 h after the first injection. CSL040 and sHuCR1 with similar glycoprofiles were generated by CSL Ltd. (Parkville, VIC, Australia) as described previously^35^. Experimental groups (n = 8/group) were as follows: (1) right nephrectomy, no left renal ischemia, no treatment (sham); (2) IRI with vehicle control (IRI/vehicle); (3) IRI with 2 doses of 15 mg/kg of CSL040; (4) IRI with 2 doses of 30 mg/kg of CSL040; (5) IRI with 2 doses of 60 mg/kg of CSL040; and (6) IRI with 2 doses of 85.2 mg/kg of sHuCR1.

### Assessment of renal function

Renal function was assessed by measuring serum creatinine using a kinetic colorimetric assay and analysed on a COBAS INTEGRA 400 plus analyzer (Roche, Castle Hill, NSW, Australia). In addition, serum urea was measured using Urea Assay Kit STA-382 (Cell Biolabs, San Diego, CA) as per the manufacturer’s instructions.

### Histopathology

Formaldehyde (10%)-fixed and paraffin-embedded kidney biopsies were cut in 4-μm-thick sections and stained with hematoxylin–eosin (H&E) or periodic acid-Schiff (PAS) according to standard protocols. PAS sections were used first to locate injured or necrotic areas, and H&E sections were subsequently used to score renal tubular injury. Injury was determined by calculating the number of injured or necrotic renal tubules as a percentage of total renal tubules within each section. Every sample was evaluated in a blinded manner by a trained pathologist for the following histological changes as findings of tubular injury: tubular epithelial cell degeneration or sloughing, tubular dilatation, cast formation, loss of brush borders, and thickening of tubular basement membranes. For each sample, twelve randomly selected corticomedullary fields (four from each of the upper, mid, and lower poles; 400× magnification) were assessed, and the percentage injury was averaged.

### Enzyme-linked immunosorbent assays for complement pathway activity, C3b, C5a, and hyaluronan

Serum samples collected 24 h after reperfusion were assessed for functional complement pathway activities using the following ELISA kits from Hycult Biotech (Uden, The Netherlands): mouse classical (HIT420), lectin (HIT421), and alternative pathway (HIT422). Plasma samples were analysed for C3b using the mouse C3b ELISA kit (HK216, Hycult Biotech) and for C5a using the Mouse Complement Activation Component C5a DuoSet ELISA kit (DY2150; R&D Systems, Minneapolis, MN) as per the manufacturer’s instructions. Plasma samples were also analysed for the glycocalyx components syndecan-1 using the mouse CD138 ELISA kit (PromoCell, Heidelberg, Germany) and hyaluronan using the DY3614 ELISA kit (R&D Systems) as per the manufacturer's instructions. OD was measured at 450 nm using a FLUOstar Omega microplate reader (BMG Labtech, Offenburg, Germany).

### Immunofluorescence

Snap-frozen kidney samples were cut into 5-μm–thick sections, air dried, and either processed immediately or stored at − 80 °C until further analysis. After fixation with acetone and hydration, the sections were stained using: mouse-anti-mouse/rat C1q Biotinylated (JL-1; Abcam, Cambridge, MA), rat anti-mouse MBL-C (14D12, Hycult Biotech), rabbit anti-mouse factor Bb (LSBio, Seattle, WA), goat anti-mouse C3d (R&D Systems, Minneapolis, MN), rabbit anti-mouse C4d (Hycult Biotech), rabbit anti-mouse C9 Alexa Fluor 488 conjugated (bs-15307R; Bioss Abs, Woburn, MA), rabbit anti-cleaved caspase-3 (5A1E, Cell Signalling Technology, Danvers, Massachusetts), rat anti-mouse VCAM-1 Alexa Fluor 488 (429; Bio-Rad, Raleigh, NC), rat anti-mouse Ly-6G FITC conjugated (1A8; BioLegend, San Diego, CA), or rat anti-mouse F4/80 FITC (A3-1; Bio-Rad). Unconjugated primary antibodies were probed with Streptavidin Alexa Fluor 594 conjugated (Thermo Fisher Scientific, Waltham, MA), goat anti-rat IgG Alexa Fluor 568 (Thermo Fisher Scientific) or goat anti-rabbit IgG Alexa Fluor 488 (Thermo Fisher Scientific). The slides were analyzed using a confocal microscope (Nikon A1R). Staining quantification was performed using Image J software version 10.2 (National Institutes of Health) as previously described^10^: fluorescence intensity by raw integrated density (RawIntDen), for C3d, C4d, C9, C1q, MBL, and factor Bb with staining throughout the tissue, by mean grey values for VCAM-1 with staining specifically in blood vessels, and by the number of Ly-6G-positive or F4/80-positive cells per high-power field (count/HPF) for neutrophil or macrophage infiltrates.

### Statistical analysis

Data plotting and statistical analysis were performed using Prism version 5.0 (GraphPad, San Diego, CA). Results are presented as mean ± standard error of the mean (SEM). To assess significance of injury between two groups (sham versus vehicle control), non-parametric tests were performed using a Mann–Whitney test. To determine the effect of treatments between 3 or more groups (different treatments versus vehicle control), non-parametric tests were performed using a Kruskal–Wallis one-way analysis of variance with Dunn’s multiple comparisons test. A p value of < 0.05 was considered to be statistically significant.

## Supplementary Information

Supplementary Figure S1.

## Data Availability

All data and material are available upon request.
