# Supplementary material for: A potent truncated form of human soluble CR1 is protective in a mouse model of renal ischemia–reperfusion injury
Source: Sci Rep. 2021 Nov 8;11:21873. doi: 10.1038/s41598-021-01423-y (PMC8575974; doi:10.1038/s41598-021-01423-y)
Supplement: Supplementary file 1 — Supplementary Figure S1. [file 41598_2021_1423_MOESM1_ESM.pdf]

A potent truncated form of human soluble CR1 is protective in a mouse model of renal ischemia-reperfusion injury

Anjan K. Bongoni[1†], Ingela B. Vikstrom[2†], Jennifer L. McRae[1], Evelyn J. Salvaris[1], Nella Fisicaro[1], Martin J. Pearse[2], Sandra Wymann[3], Tony Rowe[2], Adriana Baz Morelli[2], Matthew P. Hardy[2‡], and Peter J. Cowan[1,4‡]

[1] Immunology Research Centre, St. Vincent's Hospital, Melbourne, VIC 3065, Australia

[2] CSL Limited, Melbourne, VIC 3052, Australia

[3] CSL Behring AG, 3014 Bern, Switzerland

[4] Department of Medicine, University of Melbourne, Melbourne, VIC 3052, Australia

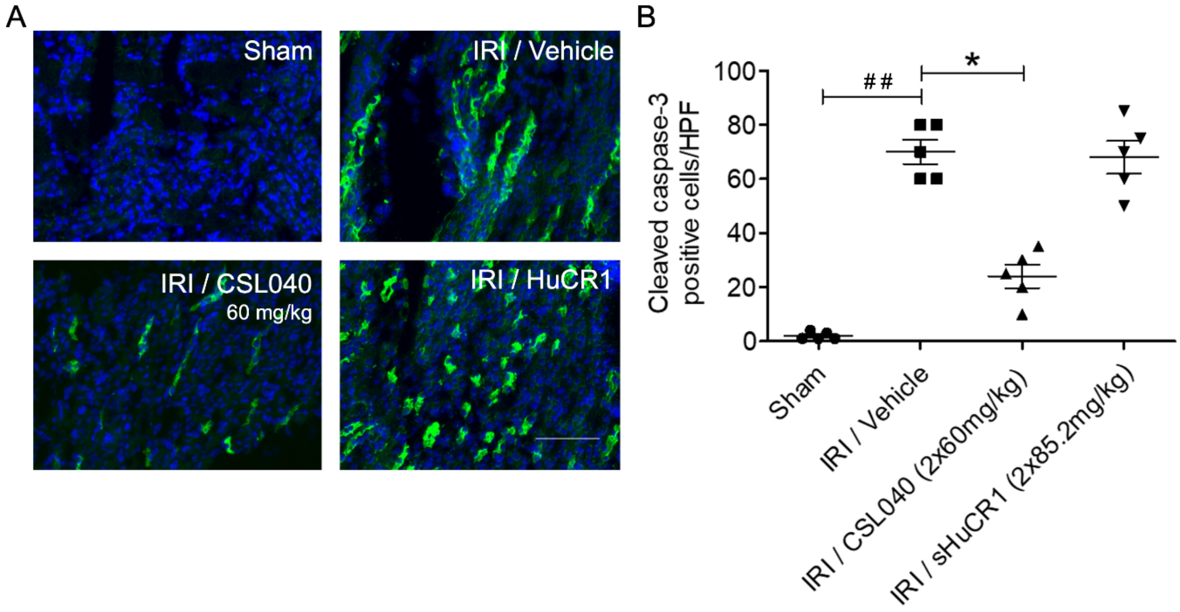

Supplementary Figure 1. Reduction of IR-induced cleaved caspase-3 positive apoptotic cells by CSL040 treatment. (A) Representative immunofluorescence images of cleaved caspase-3 positive apoptotic cells in the kidney. Scale bar: 50 μm. (B) The numbers of cleaved caspase-3 positive cells were expressed as count per high-power field (HPF), and quantitatively measured by image J analysis. Significance was tested using Mann-Whitney U test ($^{\#\#}p < 0.01$ for sham vs. IRI/vehicle), and One-way ANOVA Kruskal–Wallis and Dunn multiple comparisons test ($^{*}p < 0.05$ for IRI/vehicle vs. IRI/CSL040). The data shown are mean ± SEM (n = 5 per group).
